# Society Meetings

**Published:** 1907-09

**Authors:** 


### SOCIETY MEETINGS.

The American Association of Obstetricians and Gynecologists will hold its twentieth annual meeting at the Hotel Cadillac, Detroit, Tuesday, Wednesday, and Thursday, September 17, 18, and 19, 1907, under the presidency of Dr. Robert Tuttle Morris, of New York. An elaborate program is in preparation and it is anticipated that the number in attendance will be greater even than usual. Dr. J. H. Carstens, of Detroit, is chairman of the committee of arrangements and, assisted by other local members, is laying out comprehensive plans for the meeting. Detroit is an accessible city as well as one of great beauty and offers great attractions for medical meetings of this kind. An invitation has been extended by Messrs. Parke, Davis and Company for the association to visit their biological laboratory between the morning and afternoon sessions of Wednesday and it is probable that the invitation will be accepted.

---

The Medical Section of the National Fraternal Congress held its annual meeting in Buffalo, August 19 and 20, 1907, under the presidency of Dr. Orson Millard, of Flint, Mich. Dr. William Warren Potter, of Buffalo, delivered an address of welcome, which was responded to by Dr. H. A. Warner, of Topeka, Kan. The president then delivered his annual address after which came the reports of the secretary, treasurer, and of several special committees. Papers were then read as follows: On the Relation of the Medical Examiner to the General Office, W. K. Harrison, Chicago; The Trend of Medical Selection, F. A. Smith, Rock Island, Ill.

Dr. George B. Stocker, of Buffalo, was chairman of the local committee of arrangements.

---

The Eighth District Branch of the Medical Society of the State of New York will hold its annual meeting Wednesday and Thursday, September 25 and 26, 1907, at Buffalo, in the Historical Society building in Delaware Park, under the presidency of Dr. DeLancey Rochester, of Buffalo.

In a circular recently issued the following information is given:

It is greatly desired that as many as possible of the members of the medical profession attend this meeting. The morning of the first day will be occupied by the business of the society, carried on by the House of Delegates. The scientific session will begin at 2 pm., Wednesday, with the address of the president. In this address the president will show the advantages of union in the state society, as well as taking up some subject of general medical interest.

It is urgently requested that all members of the profession who are not members of their county society, immediately make application for membership, and that all, whether members or not, attend this meeting of the Eighth District Branch.

It is further requested that all members of the county societies, who have interesting topics for discussion, will please send the subject which each may desire to present, to the secretary of the branch before August 25th.

One hour at the end of the morning session of the second day will be set aside for the presentation of clinical cases and pathological specimens. We hope to be able to furnish facilities for the examination of cases by those especially interested. In this way it will be possible to secure a thorough discussion by different individuals of cases of doubtful diagnosis or unusual interest.

The time of the meeting is limited and it is necessary that all subjects to be presented be in the hands of the secretary by August 25, so that a proper arrangement and distribution of papers to the representatives of the several counties may be made.

The complete program will be issued early in September.

Send all communications to Lee M. Francis, M.D., secretary, 482 Delaware avenue, Buffalo.

---

The Mississippi Valley Medical Association will hold its 33d annual meeting at Columbus, O., October 8, 9 and 10, 1907, under the presidency of Dr. H. Horace Grant, of Louisville. The orator in medicine will be Dr. George F. Butler, of Chicago, and the orator in surgery, Dr. Frank D. Smythe, of Memphis.

The association is offering a prize of $100 for the best original essay upon some medical or surgical topic. The committee of the association to decide upon this contest is composed of Drs. Hugh T. Patrick, of Chicago; C. H. Hughes, of St. Louis, and A. H. Cordier, of Kansas City.

---

The American Public Health Association will hold its thirty-fifth annual meeting at Atlantic City, September 30 to October 4, 1907. under the presidency of Dr. Domingo Orvananos, of the city of Mexico. Dr. Charles O. Probst, of Columbus, is the secretary.

---

The Sixth International Dermatological Congress will be held at New York, beginning September 9, 1907, and continuing for one week, under the presidency of Dr. James C. White, of Boston. Dr. Grover W. Wende, of Buffalo, is a member of the organisation committee, and Dr. John A. Fordyce, 80 West 40th street, New York, is the secretary-general.

---

The Lake Keuka Medical and Surgical Association, at its recent annual meeting held at Grove Springs, elected the following named officers for the ensuing year: president, Louis W. Rose, of Rochester; vice-president, C. R. Jennings, of Elmira; secretary and treasurer, H. B. Nichols, of Pulteney.

